# Utilization of Composts for Adsorption of Methylene Blue from Aqueous Solutions: Kinetics and Equilibrium Studies

**DOI:** 10.3390/ma13092179

**Published:** 2020-05-09

**Authors:** Remigio Paradelo, Khaled Al-Zawahreh, María Teresa Barral

**Affiliations:** 1 CRETUS Institute (Cross-Research in Environmental Technologies), Soil Science and Agricultural Chemistry Department, University of Santiago de Compostela, 15782 Santiago de Compostela, Spain; kzawahreh@yahoo.com; 2 Department of Earth Sciences and Environment, Faculty of Natural Resources and Environment, The Hashemite University, Zarqa 13133, Jordan

**Keywords:** organic waste, waste management, waste water, colored compounds, adsorption

## Abstract

Utilization of composts as low-cost adsorbents is an important application in the field of environmental remediation, but these materials have not yet been extensively used for dye removal. In this work, we have studied the characteristics of adsorption of methylene blue onto two composts (a municipal solid waste compost and a pine bark compost). Kinetics and equilibrium batch experiments testing the influence of adsorbent particle size, solution pH and ionic strength were performed. Both composts have a high adsorption capacity for methylene blue, similar to other low-cost adsorbents. Kinetics of adsorption followed a pseudo-first-order model, with maximum adsorption reached after a contact time of two hours. Equilibrium adsorption followed a Langmuir model in general. Reduction of particle size only increased adsorption slightly for composted pine bark. Increase in ionic strength had no effect on adsorption by municipal solid waste compost, but increased adsorption by composted pine bark. Modification of pH between 5 and 7 did not influence adsorption in any case. Overall, the results suggest that electrostatic interaction between the cationic dye and the anionic functional groups in the composts is not the only mechanism involved in adsorption. In conclusion, the use of composts for dye removal is a likely application, in particular for those composts presenting limitations for agricultural use.

## 1. Introduction

Compost is the result of the aerobic biological decomposition of organic matter present in wastes by the activity of mesophilic and thermophilic microorganisms [1]. Many types of composts can be produced from a wide range of waste materials and by-products, such as urban wastes or agroindustrial wastes, and their elaboration usually requires little processing so it can be achieved with low costs [2]. Typically, composts are safe stable products, often with neutral or slightly alkaline pH, highly porous and rich in organic matter. Their main applications are in the field of agronomy and take advantage of their high organic matter content, so that they are mostly used as organic amendments in agricultural and horticultural soils [3,4,5,6] and as components in soilless horticultural substrates [7,8,9]. In addition, composts present a diversity of functional groups that are able to interact with organic and inorganic compounds, and contain microorganisms that are able to achieve biodegradation of many organic substances. This often results in a reduction of mobility and bioavailability of pollutants in soils amended with compost, in particular in the case of heavy metals [10,11]. For this reason, it is advantageously used for soil remediation processes, where composts can be employed as amendments for pollutant immobilization [12,13,14,15,16].

This ability of composts to interact with different organic and inorganic substances is the basis for another application in environmental remediation: the use of compost for pollutant removal from wastewaters, which has been investigated mainly for inorganic pollutants such as heavy metals and arsenic but also for organics such as pesticides [17,18,19,20,21]. The processes of removal of pollutants from water by composts occur through adsorption processes, and in this sense, composts can be used for wastewater treatment in the same way as other adsorbents such as activated carbon. Compared to other adsorbents, composts present advantages such as their lower cost, since they are often produced from wastes and are widely produced and available. The use of composts in environmental remediation processes is particularly interesting for those composts that present important limitations for agronomic purposes (i.e., low nutrient contents, high salinity, phytotoxicity or high levels of metals).

A potential field of application of composts as adsorbents is in the treatment of wastewater polluted with organic dyes, used extensively in the plastic and textile industries. Dye removal from wastewater is a complicated mission because of some properties of these molecules, in particular high water solubility, stability, color fastness and resistance to degradation [22]. As a consequence, they are not easily degraded under the conditions of biological treatment plants. Treatment of colored waters by adsorption processes to remove dyes has been extensively studied for low-cost adsorbents such as industrial and agricultural wastes, biomolecules such as chitosan or peats, among others, and several works have reviewed this application [22,23,24,25]. In contrast to the large number of works that have investigated the use of other types of waste material, few works have examined this potential application for composted materials. The low number of existing studies highlights the unexplored potential of adsorption onto composts as a dye removal technology and the need for further investigation regarding the factors that influence their performance or the role of composts properties on the dye removal process [26].

In order to advance in this direction, in this work we have studied the potential use of two composts for the removal of methylene blue from water. Methylene blue, known as Basic Blue 9, is a cationic dye widely used for coloring paper, cotton, silk, wool and hair. We have performed kinetics and equilibrium studies in batch experiments, where we assessed the influence of factors such as ionic strength, pH and particle size of the compost on the process of adsorption.

## 2. Materials and Methods

### 2.1. Composts

Two organic waste composts were used in the experiments. Municipal solid waste compost (MSWC) was provided by an industrial composting facility located in Galicia (Spain), and it was produced by composting source-separated organic fraction of municipal solid waste. Composted pine bark (CPB), produced through aerobic composting of pine bark in windrows, was supplied by the company Costiña Orgánica (A Coruña, Spain). The composts have been selected for this study because they present different limitations for agronomic use: in particular, MSWC presents excessive salinity and heavy metal contents, whereas CPB is acid and poor in plant nutrients, as shown in Table 1. Since it is produced exclusively from pine bark, without addition of any other material, CPB presents properties that are unusual in other types of composts, in particular its low pH and nutrient concentrations and its organic matter content close to 100%.

The functional groups of the composts were detected and identified by Fourier-transformed infrared spectroscopy (FTIR) analysis using a PerkinElmer IR instrument (PerkinElmer Dynascan Interferometer AVI, Waltham, MA, USA). For this, composts were finely ground and examined within the range 400–4000 cm^−1^. The spectra were reported as an average of 16 successive scans and they were provided in % transmittance mode.

### 2.2. Dye

Methylene blue was purchased from Panreac, S.A. (Barcelona, Spain). The dye was used as commercial salt without further purification. A methylene blue stock solution (10,000 mg L^−1^) in deionized water was prepared and subsequently diluted to the required concentrations.

### 2.3. Kinetics of Adsorption

For the study of adsorption kinetics, 500 mg of each compost (<5 mm) were weighed in polypropylene centrifuge tubes with 10 mL or 50 mL of a 200 mg L^−1^ methylene blue solution. Suspensions were shaken at 60 rpm on a rotary shaker at room temperature (25 ± 1 °C) for different times (1, 2, 4, 8, 16, 24 and 48 h), and centrifuged at 4000 g for 5 min. Aliquots from the supernatant were analyzed immediately for methylene blue by measuring absorbance at 665 nm in a UV/VIS spectrophotometer (Varian Cary 100, Agilent Technologies, Inc., Santa Clara, CA, USA). The amount of methylene blue adsorbed (mg g^−1^) was calculated as the difference between the amount of dye added initially and the amount of dye that remains in the solution at the end. Blanks without compost were run in parallel in order to test dye adsorption to the tubes. All the experiments were performed in triplicates.

Adsorption kinetics data were described using the Lagergren’s pseudo-first order and pseudo-second order models, defined by Equations (1) and (2), respectively:

(1) $$q_{a}=q_{e}\left(1-e^{-k_{1}t}\right)$$

(2) $$q_{a}=\frac{k_{2}\;q_{e}^{2}\;t}{1+k_{2}\;q_{e}\;t}$$

where *q_a_* (mg g^−1^) is the amount of dye adsorbed by the compost at a time *t*, *q_e_* (mg g^−1^) is the amount of dye adsorbed by the compost at equilibrium (this parameter is fitted by the model), *k_1_* (h^−1^) is the pseudo-first-order rate constant, and *k_2_* (g mg^−1^ h^−1^) is the pseudo-second-order rate constant.

### 2.4. Adsorption Curves

#### 2.4.1. General Procedure

For the study of adsorption, a contact time of two hours was selected following the results of the kinetics studies. For the experiments, 500 mg of each compost were weighed in polypropylene centrifuge tubes with 50 mL of solutions with different concentrations of methylene blue (100–5000 mg L^−1^). Suspensions were shaken on a rotary shaker at 60 rpm at room temperature (25 ± 1 °C) for 2 h and centrifuged at 4000 g for 5 min. The supernatant was removed and analyzed immediately for methylene blue. The amount of dye adsorbed was calculated as the difference between the dye added initially and the amount that remains in the solution at the end. All the experiments were performed in triplicates. Blanks without compost were run in parallel in order to test dye adsorption to the tubes.

Methylene blue adsorption was described using the Freundlich (Equation (3)) and Langmuir models (Equation (4)):

(3) $$q_{e}=K_{F}{C_{eq}}^{1/n}$$

(4) $$q_{e}=\frac{q_{m}K_{L}C_{eq}}{1+K_{L}C_{eq}}$$

where *q_e_* (mg g^−1^) is the amount of dye retained by the compost at equilibrium; *C_eq_* (mg L^−1^) is dye concentration in solution at equilibrium; *K_F_* (L^n^ mg^1−n^ g^−1^) and *1/n* (dimensionless) are the Freundlich coefficients; and *K_L_* (L mg^−1^) and *q_m_* (mg g^−1^) are the Langmuir coefficients.

#### 2.4.2. Effect of Ionic Strength

In order to test the effect of ionic strength, adsorption curves were prepared following the general procedure, but with methylene blue solutions prepared in 1.0 M KCl as background electrolyte.

#### 2.4.3. Effect of pH

The effect of pH was assessed preparing adsorption curves as explained in the general procedure, but the pH of the suspensions was modified by adding the necessary volume of 1 M NaOH or 1 M HCl.

#### 2.4.4. Effect of Particle Size

In order to test the effect of compost particle size on adsorption, composts were ground to pass through an 1-mm mesh sieve and then adsorption curves were performed following the general procedure.

### 2.5. Desorption

For the study of desorption, 500 mg of each compost were suspended in 10 mL of a solution with 5000 mg L^−1^ of methylene blue, in polypropylene centrifuge tubes. Suspensions were shaken on a rotary shaker at 60 rpm at room temperature (25 ± 1 °C) for 2 h and centrifuged at 4000 g for 5 min. The supernatant was removed and the centrifuged residues were weighed to calculate the amount of dye solution occluded in the solid. Then the composts were resuspended in 10 mL of water, shaken again and centrifuged as described previously and the supernatants removed. This procedure was repeated for a total of three desorption steps. Supernatants were analyzed immediately for methylene blue as explained above. Desorption was expressed as percentage of the previously absorbed methylene blue.

### 2.6. Statistics and Modelling

Modelling of adsorption isotherms and kinetics was performed by nonlinear regression analysis. The R statistical software version 3.6.1 [27], R Commander [28] and the nlstools package for R [29] were used to fit the kinetic and adsorption models to the experimental data and to calculate the significance and quality of fit of the models.

## 3. Results and Discussion

**Figure 1:**
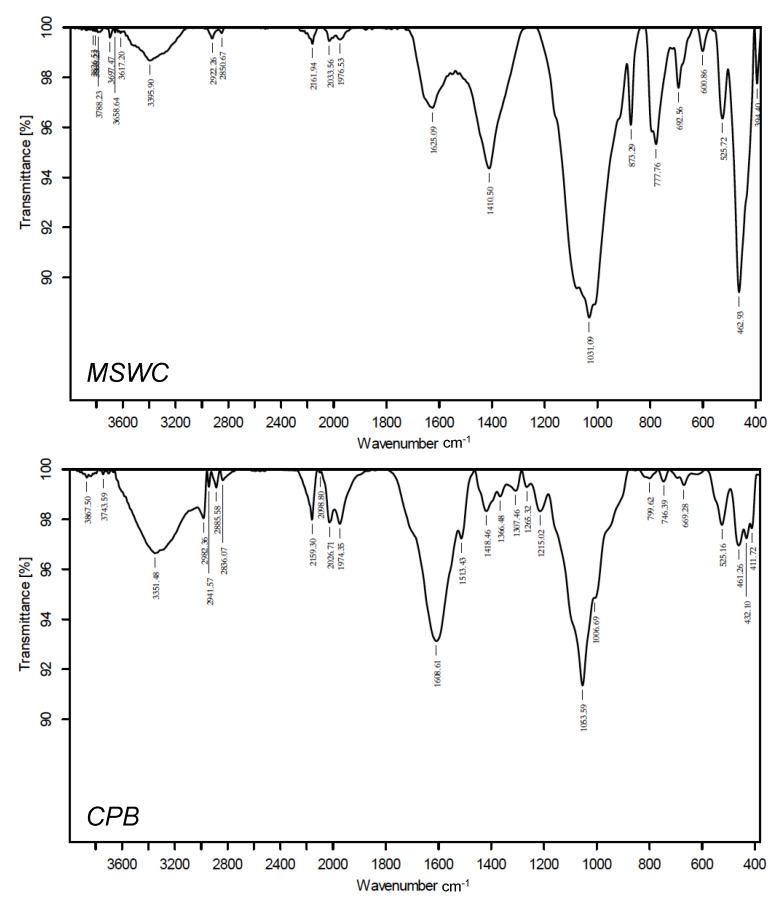
Fourier-transformed infrared spectroscopy (FTIR) spectra of the composts. MSWC: municipal solid waste compost; CPB: composted pine bark.

Figure 1 shows the FTIR spectra of the composts. Both materials presented wide bands in the 1000 cm^−1^ and 1600 cm^−1^ regions, which correspond to functional groups potentially involved in cationic dye retention. The bands at 1050–1030 cm^−1^ are attributed to C-O stretching of alcohol and sulfoxides groups, carbohydrates, polysaccharides, as well as clay minerals in the case of MSWC. The bands in the region around 1500–1600 cm^−1^ can be assigned to amide, carboxylates, and C=C in the aromatic rings of lignin and lignocellulose. CPB shows an intense peak at 1608 cm^−1^ while MSWC has a smaller IR peak at 1625 cm^−1^. This peak is higher in CPB than in MSWC due to the higher lignin content in the first. MSWC showed an intense band at 1410 cm^−1^ while CPB showed a much weaker band at 1418 cm^−1^. This peak is attributed to nitrates and reflects the higher N content in MSWC with respect to CPB. Both composts present bands corresponding to vibration of O-H stretch of hydroxyl groups and water (around at 3351 cm^−1^ for CPB and 3395 cm^−1^ for MSWC) and (–CH2–) stretching of methylene groups in the 2800–2900 cm^−1^ region. The peak at 873 cm^−1^ in MSWC is attributed to carbonate and is absent in CPB, which has acid pH and lacks these components.

**Figure 2:**
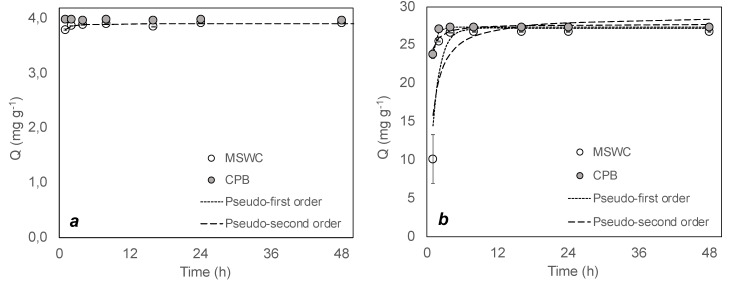
Kinetics of adsorption municipal solid waste compost (MSWC) and composted pine bark (CPB) at (**a**) 1:20 solid:liquid ratio and (**b**) 1:100 solid:liquid ratio. Bars show standard deviation of the mean (n = 3).

Figure 2 show the kinetics of adsorption for MSWC and CPB at two solid:liquid ratios. Percentages of adsorption close to 100% were obtained in all cases. With 1:20 solid:liquid ratios, adsorption equilibrium was reached after one hour for CPB and after two hours for MSWC. When using the 1:100 solid:liquid ratios, adsorption was slower: MSWC required four hours for reaching the maximum, whereas CPB required two hours. This suggests that equilibrium time and adsorption rate are not independent from the amount of available dye. Kinetics is faster at low methylene blue charges, as shown in our own results: kinetics was slower at the solid/liquid ratio (1:100), which implies a 5-fold increase in the amount of methylene blue per mass of compost with respect to the 1:20 ratio. Some works have found that maximum adsorption values for methylene blue by compost are reached in some cases in a matter of minutes [30,31], whereas other studies have found equilibrium times of 3–8 h [32,33]. The latter are in better agreement with compost adsorption kinetics of other basic dyes, such as Basic Violet 10, Basic Green 4 or Basic Violet 16, which have equilibrium times of 1–5 h [34,35,36,37]. Some of these works report a decrease of adsorption rate of basic dyes by composts with increasing dye concentrations [36], in agreement with our observations, so it is likely that the incongruences found in the literature for equilibrium time are due to divergences in dye/adsorbent ratio employed in the experiments.

Fittings of kinetic models to the experimental data are shown in Table 2. The values of the parameters confirm that the rate of adsorption depends on the amount of dye available to be adsorbed, with constants that increase at a lower solid:liquid ratio in both models. Our data show that the pseudo-first-order model fits better to the experimental results, in contrast with most studies of methylene blue adsorption on various sorbents, which generally find that kinetics follows a pseudo-second order model [38]. In the specific case of methylene blue adsorption by compost, the existing studies do not report fitting of kinetic models, in some cases due to excessive quickness of adsorption [31]. Studies of compost removal of other basic dyes also show good fit of pseudo-second order model [35,36,37], but it is not clear that it is superior to pseudo-first-order model. In particular, data obtained by Bhagavathi et al. [36] for malachite green show that when high loads of dye are used, first-order kinetics explain better the results and produce better predictions of the maximum adsorption capacity.

**Figure 3:**
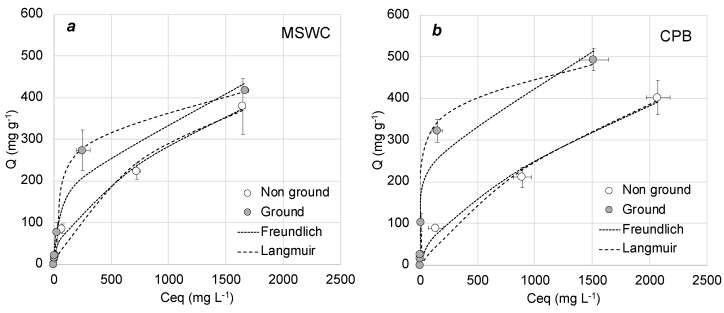
Adsorption curves at different particle size for (**a**) municipal solid waste compost (MSWC) and (**b**) composted pine bark (CPB). Bars show standard deviation of the mean (n = 3).

**Figure 4:**
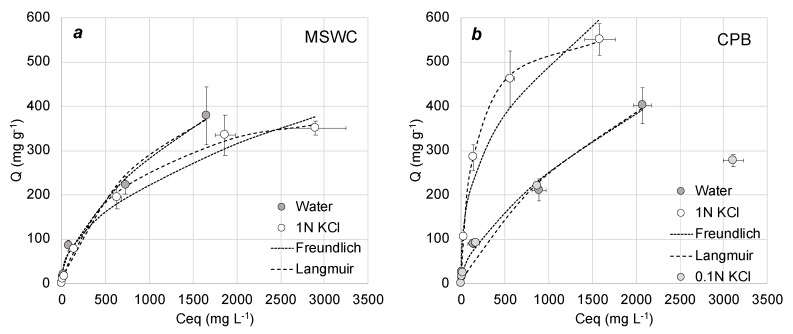
Adsorption curves in presence and absence of 1 M KCl for (**a**) municipal solid waste compost (MSWC) and (**b**) composted pine bark (CPB). Bars show standard deviation of the mean (n = 3).

**Figure 5:**
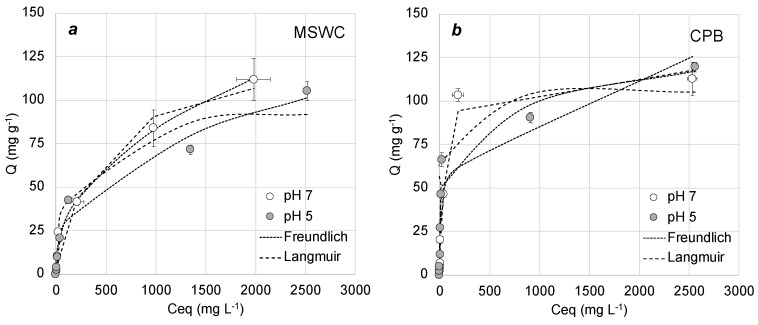
Adsorption curves at different pH for (**a**) municipal solid waste compost (MSWC) and (**b**) composted pine bark (CPB). Bars show standard deviations of the mean (n = 3).

Figure 3, Figure 4 and Figure 5 show the adsorption curves of methylene blue onto composts under different conditions, whereas Table 3 shows the results of fittings to Freundlich and Langmuir adsorption models under all conditions. Overall, adsorption was better described by the Langmuir model (which assumes a maximum capacity of adsorption and no interaction between adsorbate molecules) than by the Freundlich model. This is in agreement with the literature on methylene blue adsorption by composts [30,31,33] and other adsorbents [38]. This behavior is also common in the adsorption of other cationic dyes by compost, which in general follows the Langmuir model [35,36,37]. Modelled maximum adsorption of the composts in our work is in the range of 400–800 mg g^−1^, depending on the experimental conditions. These values are slightly higher than those found in similar studies with compost: for example, Anastopoulos et al. [31] and Baghavati et al. [30] obtained maximum adsorption capacities of 250 and 296 mg g^−1^ for olive waste compost and water hyacinth compost, respectively. In addition, the values found here are comparable to the best low-cost adsorbents available in the literature, such as activated carbons or biochars [38,39]. Since methylene blue is a cationic molecule and therefore electrostatic interaction can be an important mechanism of adsorption, it is interesting to note that their maximum adsorption capacity would be approximately ten times higher than their cation exchange capacity, as shown in Table 1. This suggests that electrostatic attraction is not the only mechanism of adsorption of methylene blue onto the composts. 

Figure 3 shows the effect of compost particle size on adsorption. In theory, a reduction in particle size of an adsorbent translates into the increase in the external surface area, resulting in more active sites for adsorption and, consequently, a more efficient adsorption. However, we did not observe a clear positive effect on the adsorption by MSWC and only a slight increase for CPB. In this sense, an increase of dye adsorption with compost size reduction is not always observed, or at least not for any size reduction. For example, Baghavati et al. [30] did not observe improvement of methylene blue adsorption with a decrease in compost size from 2.5 to 0.5 mm. Since composts are materials with high porosity and large internal surface area because of their high content in organic matter, it is likely that adsorption is not limited by particle size, which determines the external surface. This would explain the lack of effect of size reduction in the range tested.

Figure 4 shows the effect of ionic strength on adsorption by presenting the adsorption curves of methylene blue in water and in 1 M KCl solution. For the compost MSWC, there was no difference nor did the adsorption constant change. For CPB, however, the adsorption increased in 1 M KCl with respect to water. In this sense, Heidarinejad et al. [40] reported that methylene blue adsorption on activated carbon showed a significant increase by increasing ionic strength from 0 to 1 M NaCl. This fact suggests again the implication of mechanisms other than electrostatic interaction in methylene blue adsorption: if electrostatic interaction were the main mechanism, high ionic strength should increase competition of other cations with methylene blue for adsorption sites and potentially reduce adsorption. According to Heidarinejad et al. [40], the increase in adsorption with ionic strength could be explained by two complementary mechanisms that do not necessarily exclude electrostatic interaction. First, increase in ionic strength would produce a compression of the diffuse double layer on the adsorbent surface, which is favorable for the electrostatic attraction. Second, higher ionic strength would increase the intermolecular forces between dye molecules, such as dipole–dipole, ion–dipole or Van der Waals dispersion forces, thus decreasing the repulsive interaction between molecules. This mechanism would favor the formation of dye aggregates in the solution that would later migrate towards the adsorbent, thus improving retention, as observed by Fedoseeva et al. [41] for the cationic dye malachite green. Also, the addition of anions could stimulate the migration of cations toward the interface favoring this aggregation. In any case, the fact that the increase of ionic strength does not influence negatively methylene blue adsorption by compost is positive for real application. Industrial wastewaters are not pure solutions of dyes in water and therefore they can present high ionic strength as well as mixtures of compounds, so reduction of performance with ionic strength would be a negative fact.

Regarding the potential effect of pH, the adsorption curves plotted in Figure 5 show that pH change between 5 and 7 did not influence methylene blue adsorption by the composts. This observation agrees with results by Anastopoulos et al. [31], who reported no effect of pH on methylene blue adsorption on olive waste compost, but it disagrees with many works in the literature, which state that the adsorption of methylene blue is higher at alkaline pH than at acid pH [38,39]. Adsorption of basic dyes by composts has also been observed to increase with pH, in agreement with this [34,35,36]. The lack of effect of pH is not compatible with electrostatic interaction being the main mechanism for methylene blue adsorption by compost. Solution pH influences both the charge of the adsorbent and the charge of ionic dyes dissolved. Basic dyes such as methylene blue generally have acid functional groups that are positively charged at pH values above their pKa, and neutral at lower pH. Since composts are negatively charged, increasing pH of the solution will produce an increase in electrostatic attraction between cationic dyes and the adsorbent surface, subsequently leading to higher adsorption. Therefore, our results show that methylene blue adsorption by composts is a complex process in which electrostatic interaction is not the main or only mechanism, in line with the implications of the influence of ionic strength on adsorption. Other mechanisms that could be involved in this process are hydrogen bonds, hydrophobic forces and π–π interactions, as observed by other authors for methylene blue adsorption onto activated carbon [42,43,44]. In particular, the large bands due to polysaccharides and aromatic rings observed in the FTIR spectra shown in Figure 1 point to an important role of hydrophobic interactions in the process.

Finally, desorption must also be taken into account as an important aspect for the use of composts as dye adsorbents. For optimal dye removal, desorption of the dye adsorbed by the compost must be minimal during operation. Table 4 shows that the desorption of methylene blue, in both composts and at different pH values, are very low (<6%), in particular for composted pine bark, that presented virtually no desorption. Other authors have also observed very low desorption by water of methylene blue adsorbed on compost [31]. Although this is a positive result that shows strong retention by the adsorbent, it would not be ideal from the point of view of potential adsorbent reuse. Regeneration of adsorbents for reuse can minimize operational costs and reduce the production of waste, although this aspect is less relevant for composts and in general low-cost adsorbents. These can be obtained in large quantities at low cost from materials of residual origin, in comparison to other adsorbents such as activated carbon, which are produced in lower quantities and at higher costs. Other options for the management of used adsorbents are more adequate in this case, for example further composting to promote biodegradation of the adsorbed dyes [45].

## 4. Conclusions

In this work we have explored the potential application of two composts (municipal solid waste compost and pine bark compost) for dye removal, by studying kinetics and equilibrium adsorption of methylene blue. Kinetics experiments showed that adsorption occurs mainly in the first 1–2 h of contact and it follows a pseudo-first order kinetic model. Equilibrium adsorption follows a Langmuir type adsorption model. It was not affected by modification of pH between 5–7 and was only slightly by reduction of compost particle size, whereas increase in ionic strength improved adsorption only by composted pine bark. The results suggest that electrostatic interaction is not the only mechanism involved in adsorption in this case. Overall, both composts are efficient adsorbents for methylene blue, with a high adsorption capacity when compared to other adsorbents, and have advantages such as their lower cost and higher availability. Given that some particular composts are not optimal for agricultural or horticultural use, their use for dye removal is an interesting application that must be further studied in the future.

## Figures and Tables

**Table 1:** Composts properties. EC: electric conductivity; OM: total organic matter; CEC: cation exchange capacity; AEC: anion exchange capacity.

Compost	pH	EC	OM	C	N	C/N	P	CEC	AEC	Cu	Pb	Zn
		dS m^−1^	%	%	%		g kg^−1^	cmol_c_ kg^−^^1^	cmol_c_ kg^−^^1^	mg kg^−1^	mg kg^−1^	mg kg^−1^
MSWC	8.5	7.01	41.8	24.3	1.57	16	14.2	19.4	3.6	1934	1065	2194
CPB	5.3	0.37	91.4	53.1	0.28	194	0.26	26.2	4.6	10	6	29

**Table 2:** Kinetic model parameters (estimation ± standard error). MSWC: municipal solid waste compost; CPB: composted pine bark. S/L: solid/liquid ratio; k_1_: pseudo first-order kinetic rate; k_2_: pseudo second-order kinetic rate; R^2^: correlation coefficient between experimental and modeled data. Significance of the parameter estimations is indicated as follows: * significant at a *P*-value of 0.05; ** significant at a *P*-value of 0.01; *** significant at a *P*-value of 0.001.

S/L	Compost	Pseudo-First Order Model				Pseudo-Second Order Model		
		k_1_ (h^−1^)	q_e_ (mg g^−1^)	R^2^	k_2_ (g mg^1^ h^−1^)		q_e_ (mg g^−1^)	R^2^
1:20	MSWC	3.6 ± 0.2 ***	3.9 ± 0.01 ***	0.77	9 ± 7		3.9 ± 0.01 **	0.80
	CPB	- ª	-	-	-		-	-
1:100	MSWC	0.8 ± 0.2 **	27 ± 2 ***	0.86	0.04 ± 0.02		28.8 ± 0.4 *	0.71
	CPB	2.0 ± 0.03 ***	27 ± 0.04 ***	0.99	0.27 ± 0.02 **		27.8 ± 0.04 **	0.80

ª data for CPB could not be adequately described by the models in this case.

**Table 3:** Adsorption model parameters under different conditions (estimation ± standard error). MSWC: municipal solid waste compost; CPB: composted pine bark. K_F_ (L^n^ µmol^1−n^ kg^−1^), 1/n (dimensionless): coefficients of the Freundlich equation; K_L_ (L mg^−1^), q_m_ (mg g^−1^): coefficients of the Langmuir equation; R^2^: correlation coefficient between experimental and modeled data. Significance of the parameter estimations is indicated as follows: * significant at a *P*-value of 0.05; ** significant at a *P*-value of 0.01; *** significant at a *P*-value of 0.001.

**Compost**	**S/L**		**Freundlich Model**			**Langmuir Model**		
			**K_F_**	**1/n**	**R^2^**	**K_L_**	**q_m_**	**R^2^**
**MSWC**	1:100	Ground	24 ± 12	0.39 ± 0.07 **	0.953	0.006 ± 0.0006 ***	454 ± 11 ***	0.998
		Nonground						
		Water	6.1 ± 1.8 *	0.55 ± 0.04 ***	0.995	0.0009 ± 0.0005	621 ± 180 *	0.982
		1 M KCl	7.9 ± 3.4	0.49 ± 0.06 ***	0.983	0.001 ± 0.0001 ***	452 ± 21 ***	0.996
	1:20	pH 5	4.9 ± 1.5 *	0.39 ± 0.04 ***	0.977	0.006 ± 0.002 *	98 ± 8 ***	0.958
		pH 7	4.1 ± 0.8 **	0.44 ± 0.03 ***	0.992	0.002 ± 0.0009 *	130 ± 16 ***	0.976
	**S/L**		**K_F_**	**1/n**	**R^2^**	**K_L_**	**q_m_**	**R^2^**
**CPB**	1:100	Ground	56 ± 21	0.30 ± 0.06 **	0.952	0.014 ± 0.007	504 ± 46 ***	0.977
		Nonground						
		Water	3.5 ± 1.7	0.62 ± 0.06 ***	0.992	0.0004 ± 0.0003	876 ± 357	0.985
		1 M KCl	33 ± 15	0.39 ± 0.07 **	0.946	0.006 ± 0.0007 ***	600 ± 19 ***	0.997
	1:20	pH 5	30 ± 4 ***	0.17 ± 0.02 ***	0.971	0.08 ± 0.03 *	106 ± 9 ***	0.948
		pH 7	15 ± 7	0.27 ± 0.07 **	0.818	0.02 ± 0.004 **	120 ± 5 ***	0.988

**Table 4:** Desorption percentages of previously adsorbed dye after contact with compost (mean ± standard deviation, n = 3). MSWC: municipal solid waste compost; CPB: composted pine bark.

**MSWC**	**Step 1**	**Step 2**	**Step 3**
pH 7	1.5 ± 1.3	5.2 ± 3.2	5.8 ± 3.2
pH 5	0.7 ± 0.8	0.5 ± 0.8	0.2 ± 0.8
**CPB**	**Step 1**	**Step 2**	**Step 3**
pH 7	0.2 ± 0.03	0.2 ± 0.02	0.2 ± 0.02
pH 5	0	0	0
